# Understanding Nuance and Ambivalence in Intergenerational Relationships Through Fiction

**DOI:** 10.1093/geront/gnad051

**Published:** 2023-04-27

**Authors:** Jade Elizabeth French, Melanie Lovatt, Valerie Wright

**Affiliations:** English Department, Loughborough University, Loughborough, UK; Faculty of Social Sciences, University of Stirling, Stirling, UK; School of Humanities, University of Glasgow, Glasgow, UK

**Keywords:** Generationalism, Literature, Novels, Reading groups

## Abstract

**Background and Objectives:**

The term “intergenerational relationships” is widely used in gerontological literature and age-related policies. However, discussions of the term often tell us surprisingly little about what it means or why it matters. We suggest that this is due to a reductivism and instrumentalism in 2 main discourses within which intergenerational relationships are usually discussed. First, intergenerational relationships are often conceptualized through a binary “conflict/solidarity” lens, reinforcing an entrenched “generationalism”. Second, they are predominantly constructed as a problem to be addressed within debates on how to tackle intergenerational segregation. Neither of these discourses provides much room for a more nuanced understanding of how intergenerational relationships are experienced or why they are meaningful. In this paper, we discuss how fictional narratives can introduce imagination and a richer vocabulary into discourses concerning how people of different ages relate to each other.

**Research Design and Methods:**

We present findings from reading groups where adults discussed novels depicting themes of older age, intergenerational relationships, and time.

**Results:**

In discussing the fictional narratives and characters, participants reflected on the significance and meaning of intergenerational relationships in ways that went beyond dichotomous and instrumentalist discourses. Drawing on the concept of lived ambivalence, we argue that fictional representations of intergenerational themes can elicit more meaningful reflections on the complexities and contradictions of relationships across age groups.

**Discussion and Implications:**

We conclude that a more nuanced understanding of intergenerational interaction can inform gerontological discourses and policy, but also that gerontological awareness of social challenges concerning age relations can inform interpretations of fictional narratives.

The language used to discuss aging has been criticized for its “poverty” (Marshall, 2022; Small, 2007, p. 3), reflecting and reinforcing instrumentalist and reductive approaches to aging that do not convey the nuances and lived experiences of a complex process lived in time (Baars, 2014). We argue that the same could be said for language used to discuss intergenerational relationships. The term has become ubiquitous across academic, policy, and media discourses, and is often used as a shorthand to describe different generational cohorts. Finding no adequate synonym, we use the term ourselves, but we seek to broaden the vocabulary and deepen understandings of what we mean by intergenerational relationships. We do so using the concept of lived ambivalence (Baars, 2014) and a creative qualitative methodology of analyzing participants’ responses to novels. We argue that novels that depict characters whose experiences and relationships are portrayed in a narrative form, over time, can be a useful way of opening up discourses of lived ambivalence. Rather than dichotomous discourses that imply a “cross-sectional” approach to intergenerational relationships, in which each generation is fixed in time, fiction can facilitate more nuanced, processual, *human*, and fundamentally more meaningful understandings of how intergenerational relationships are lived in time and what they mean to people.

## Intergenerational Dichotomies

We argue that there are two main discourses of intergenerational relationships, both of which reinforce a sense of rigid generational boundaries, instrumentalism, and reductivism, and neither of which are helpful in facilitating a more nuanced understanding of relationships between people of different ages. The first discourse can be understood as “generationalism,” a mindset that “instigates artificial confrontations between the ‘generations’” (Purhonen, 2015, p. 102) in a simplified and exaggerated way, perpetuating a narrative of generational injustice and ongoing crisis that divides generations (White, 2013, p. 217). This discourse relies on a conflict/solidarity binary that frames intergenerational relationships as sites of antagonistic, zero-sum battles between different generational cohorts, most currently typified as “boomers” and “millennials.” It is often found in the media and social media, where “the assumption of an economic and political conflict between the two generations has become an established trope” (Bristow, 2021, p. 766). These conflict narratives often invoke the future, with calls for “intergenerational justice” (Tremmel, 2006) to address what are framed as future threats caused to today’s children and younger people by today’s “older people.” In response to this, there have been calls for a greater “intergenerational solidarity” that puts generational divisions aside and comes together for the sake of a more broadly imagined humanity (UN Secretary-General, 2013). The conflict/solidarity discourse is related to a second dichotomous discourse that identifies “intergenerational segregation” as a problem that needs to be addressed through increased intergenerational integration (Kingman, 2016). Examples of this include interventions and calls for policies intended to facilitate greater intergenerational interaction in housing (Hoolachan & McKee, 2018; Kingman, 2016), educational settings (Boström & Schmidt-Hertha, 2017; George et al., 2011), workplaces (Lagacé et al., 2019), and communities in general (Zhong et al., 2020).

A desire for greater intergenerational solidarity and integration is understandable, particularly as intergenerational relationships have been centered on discourses concerning several recent political and environmental issues (Bristow, 2021; Elliott, 2021; Rios et al., 2021). However, seeking solidarity in intergenerational relationships can be limiting as it requires a consensus that minimizes the diversity of different experiences (Bengtson & Oyama, 2010; Connidis & McMullin, 2002) and risks ignoring how age intersects with other social divisions such as class, gender, race, and ethnicity (Elliott, 2021; White, 2013). An uncritical emphasis on solidarity can paper over the tensions and contradictions inherent in personal and social relationships (Davies, 2021) and render the term “intergenerational relationships” anodyne. To gain a more meaningful understanding of why and how intergenerational relationships matter to us, we must go beyond dichotomous discourses that mask the messiness of lived relationships, to find a way to expand the discourses of how people of different ages relate to each other. We argue that the concept of ambivalence can be helpful in this regard.

## Ambivalence and Intergenerational Relationships

Ambivalence has been used in aging studies to cut through simplistic dichotomies, for example, in understanding conflicting feelings toward the future in people living with dementia (Thuessen & Graff, 2022). It has also been used in research on intergenerational relationships as a “bridging concept between social structure and individual action” (Connidis & McMullin, 2002, p. 559), that also has a “dynamic, transformative and temporal dimension” (Hillcoat-Nallétamby & Phillips, 2011, p. 214). As such, ambivalence allows for differences of opinion and reflects the way personal relationships can often be “sticky” and difficult to “shake free from… at an emotional level” (Smart, 2007, p. 45). Even when conflict is present around topics where values may differ (e.g., environmental concerns, care, policies, etc.) relationships still survive (Davies, 2021). Defining ambivalence as the coexistence of mixed or contradictory emotions, attitudes, or ideas about the same person, object, or situation by an individual subject, it can offer “a mature step toward acknowledging a more complex world of multiple perspectives and emotional resilience” (Biggs, 2007, p. 706). Lüscher (2011) conceptualizes “ambivalence” as a useful counterpoint to a desire for solidarity which, while appealing, can imply an idealized and static worldview that can ignore function and process (p. 194). The concept of ambivalence allows us to get closer to how intergenerational relationships are *lived* within kinship and social networks.

The significance of aging as a process and of how relationships are *lived* coheres with calls from scholars of aging studies to draw on approaches from the humanities to better understand these “deeply humane processes and experiences” (Baars, 2014, p. 46). Baars argues that our experiences of living and aging through time cannot be reduced to the “either-or dichotomies” (p. 45) he identifies as being prevalent in academic and public discourses on aging, and so advocates for a “lived ambivalence” that can cut through these. One way to better understand the complexities of aging is through cultural and literary texts. Free from the conventions of policy-making and scientific research that often reduce human experience to empirical data (p. 45), fiction is better equipped to explore the ambiguities, contradictions, and richness of the relational and processual nature of aging.

There is a substantial body of work by humanities scholars who draw on fictional texts to better understand aging as a process and experience (see e.g., Small, 2007; Woodward, 2019) demonstrating how aging is a lifelong process informed by cultural, social, and material forces (Kriebernegg et al., 2014). Fiction has also been used by health care professionals in geriatric medicine to communicate and explore the emotional aspects of aging with patients (Roitto & Rognstad Mellingsæter, 2019). Sociologists have used fiction as an alternative source of knowledge to provoke new perspectives on the social world and as a “spark for theoretical rumination” (Beer, 2016, p. 410). Within literary studies, Felski (2019) has argued that identification with fictional characters can provide access to shared experience without negating difference and enhance a reader’s sense of self in society. Alongside character, narrative emotion encourages readers to become empathetic co-creators of fictional worlds by “respond[ing] to the techniques of storytelling with curiosity, suspense and surprise” (Keen, 2015, p. 152). A growing body of work in the social sciences and literary studies demonstrates the value of using literary criticism as an empirical device to investigate social issues and ambivalences (Suckert, 2021) and to elicit lay reflections on cultural narratives of aging, addressing a perceived hierarchy and disconnect between professional and “ordinary” readers (Swinnen, 2023). The relationship between novelists and aging scholars also works the other way; the British author Margaret Drabble acknowledges that her novel *The Dark Flood Rises* was partly inspired by Helen Small’s *The Long Life* (Drabble, 2017, p. 326).

There is less of a tradition of using literary texts to understand the diversity and complexity of intergenerational relationships in gerontology, yet there is scope to use fictional representations of intergenerational relationships to relate and understand their social impacts. Literary texts have been used to facilitate and study intergenerational relationships, be that exploring the nature of play and connectivity (Deszcz-Tryhubczak & Kalla, 2021) and highlighting the mutual learning benefits that emerge in intergenerational reading groups (Lohman et al., 2003). Intergenerational reading groups offer a fruitful ground for analyzing complex attitudes to relationships between people of different ages, as together readers make meaning both individually and as a collaborative group, drawing on experiences across life spans.

## Methodology

The findings presented here are part of an Economic and Social Research Council-funded project, Reimagining the Future in Older Age, which aimed to examine the relationship between older age and future time. The project received ethical approval from the University of Stirling. We undertook a qualitative study that brought together adults of different ages to analyze and reflect on dominant narratives of aging and intergenerational relationships by discussing novels that depicted these themes. We chose novels as a literary form because of their accessibility to a range of readers who might differ in confidence, and because we judged their typical length and narrative structure conducive to portraying aging and relationships as processes, in keeping with the project’s focus on temporality.

The project team drew up a long list of novels, which participants were then invited to choose from and add to. Only one novel was selected solely by participants. The novels were primarily chosen because they depicted themes of aging, time, and intergenerational relationships that were central to the project. We purposively sought novels from a range of genres and eras in order to represent different intergenerational relationships—from the historical to the contemporary and from the realist to the speculative—to give readers a range of imagined societies, life spans, and fictional world-building scenarios to respond to, sparking imaginative conversations about readers’ own experiences. For more information on the novels and the intergenerational relationships foregrounded, please see Supplementary Material. Reading groups and book clubs have been found to encourage rapport and relational links, particularly in an intergenerational context where adults of all ages can develop a “mutually advantageous” understanding of one another’s situation (Lohman et al., 2003, p. 104). Using literature as a creative method provided a way to engage with participants’ lived experiences as they compared their social worlds with those in the novels. Specific genres, such as speculative fiction, are driven by a goal that returns us “to our own [world] enlivened with deeper understanding and insight… and fresh perspectives on how to build our age world in meaningful and just ways” (Woodward, 2019, p. 367). As such, fiction can be used as a provocation to prompt thoughts, ideas, and feelings on real-life topics as well as a springboard to imagine different solutions and scenarios in the real world.

Although originally planned as face-to-face meetings, due to coronavirus disease 2019 (COVID-19), the reading groups were facilitated online by M. Lovatt and V. Wright, and this digital element allowed us to recruit participants from across central Scotland. Participants were recruited via social media and the project website and through snowball sampling. In an attempt to recruit a diverse sample of participants across different social characteristics, we also sent targeted invitations to a range of community and third-sector organizations representing minority groups in Scotland. Despite this, the majority of participants were white women—a bias that reflects the typical composition of reading groups (Tepper, 2000). Reflecting on our recruitment strategy in meetings with our project advisory group, we wondered if we might have been more successful had we been able to make in-person visits to organizations representing minority groups in order to explain the project in more detail, build relationships and elicit suggestions for novels; however, these opportunities were denied us due to social distancing regulations in place at the time. In the end, 28 participants were recruited to four reading groups, although not everyone attended all of the discussions due to a range of reasons including changing commitments and personal circumstances related to the pandemic. We tried to ensure age diversity in each group (see Table 1). The groups met monthly from June to October 2020. Each group was made up of adults of different ages to allow for reflections on the novels from a range of perspectives.

**Table 1.** Reading Groups, Participants, and Novels Read

Reading group	Novels read	Name (pseudonym)	Gender	Age
Group 1	*Trouble With Lichen, Young Art and Old Hector Last Children of Tokyo, Never Let Me Go*	Jane	Female	69
		Frankie	Female	60
		Oliver	Male	53
		Kirsty	Female	36
		Rachel	Female	29
		Duine	Male	71
		Clare	Female	23
		Jen	Female	40
Group 2	*The Summer Book, Never Let Me Go, Last Children of Tokyo, Moon Tiger, The Dark Flood Rises*	Katherine	Female	76
		Rose	Female	68
		Florence	Female	55
		Rebecca	Female	48
		Gillian	Female	28
		Jonathan	Male	21
Group 3	*The Summer Book, Trouble With Lichen, Last Children of Tokyo, The Sixteen Trees of the Somme, Dark Flood Rises*	Brian	Male	76
		Greta	Female	70
		Sanny	Male	65
		Elizabeth	Female	60
		Christine	Female	53
		Ashleigh	Female	37
		Jamie	Male	29
Group 4	*The Summer Book, Trouble With Lichen, Turnabout, The Sixteen Trees of the Somme, Young Art and Old Hector*	Agnes	Female	58
		Fiona	Female	42
		Jess	Female	30
		Michelle	Female	39
		Angela	Female	39
		Eleanor	Female	62
		Kitty	Female	70

The reading group sessions lasted approximately 90 min and usually began with the participants and researchers checking in with each other, providing a type of informal emotional support network as we shared how we were feeling under lockdown conditions and as things began to open again. We then invited participants to give their initial reflections on the novel, followed by a semistructured conversation based on prompting questions circulated in advance. The questions were based on M. Lovatt’s and V. Wright’s readings of the novels and related to the research interests of aging, time, and intergenerational relationships. Although the questions were useful as a starting point, discussions often spiraled out to a range of related topics raised by participants, and they shared their own stories and experiences outside of the reading group. All discussions were video- and audio-recorded and transcribed verbatim. Participants were further invited to keep diaries in which they could record their thoughts on the novels outside of the discussions and situate the readings in the contexts of their everyday lives and any other reflections they thought were relevant.

The data comprised transcripts from 19 reading groups and 43 diary entries (1 group only met four times but discussed five novels). In the first stage of analysis, V. Wright and M. Lovatt read the transcripts, uploaded them to NVivo 13, and conducted open coding, which resulted in several themes of relevance to the project. J. E. French then led a further round of analysis which focused on the theme of intergenerational relationships and which was guided by the question “How can fiction expand our understanding of intergenerational relationships?.” As J. E. French was not present for the data collection, she supplemented her analysis of the transcripts with watching video footage of the groups and sharing interpretations of the data with the other authors. J. E. French refined the themes using a mind-mapping approach to create a thematic map of the data and to further search for relevant patterns using an inductive analysis approach. This flexible approach was taken due to the breadth and depth of the discussions, in order to capture as much richness of the conversations as possible. That the analysis was conducted by researchers who were present during data collection and a researcher who was not, increased our confidence that our claims to knowledge were justified, and yielded insights that we may not have otherwise had, in a manner similar to what James (2013) calls “the analytic imagination.” Not all novels are featured in this paper, reflecting this paper’s focus on intergenerational relationships rather than aging and time more broadly, which are covered in the other novels.

## Findings

Our analysis identified three key ways participants’ discussions opened up new understandings of intergenerational relationships: (1) a greater understanding of what intergenerational relationships are, who they involve and what they ought to be; (2) a more nuanced understanding of emotional connection between generations; and (3) a desire for more “authenticity” in fostering intergenerational interaction.

### A Greater Understanding of What Intergenerational Relationships Are

While the novels featured relationships between characters of different ages, none of these were explicitly defined as “intergenerational relationships.” The fictional representations opened up lines of communication with the “generational other” (Biggs, 2007, p. 708) and the narration of complex characters entangled in relationships in a range of scenarios perhaps helped participants to talk about relationships in ways that transcended dichotomies. This was revealed in the ambivalence that participants expressed about their own relationships with people of different ages (particularly in the context of care), how they felt intergenerational relationships might change with increased life expectancies, and the relationships between ancestors and descendants.

Intergenerational caring relationships within families were discussed in relation to *The Last Children of Tokyo*, a dystopian speculative fiction novel depicting the relationship between 108-year-old Yoshiro, who has an increased vitality and life expectancy, and his ailing great-grandson, Mumei, who will die young because of a variety of environmental and social disasters. Participants reflected on the extended life expectancy of the older characters in the novel and the implications of this for the nature of intergenerational relationships. Jen (40, G1) said “I think it ... challenges us to think about what we mean by grandparents, great-grandparents ... I think sometimes when you use the words like grandparents and great-grandparents we all have an image in our mind of what we assume that person to be, that age to be, but there’s such a variety in society nowadays.” Participants felt that the greater involvement of great-grandparents in caring for younger relatives was not unlikely, with Kitty (70, G4) suggesting:

this might be the first generation where it’s not unreasonable to assume that great-grandparents will have a caring role with their great-grandchildren ... my grandchildren range from 18 months to 30 years old ... I think for me the kinda realisation that great-grandparents now can be looking forward to actually having active roles ... I hadn’t thought about that before.

When asked about what she felt about this extension of caring responsibilities, she expressed ambivalence, saying, “I have to be honest ... deep down you want to help your children but there is a bit where you think ‘I’ve already done this’ ... obviously there’s lovely bits to it but physically I don’t feel equipped really.” She later added that while she was still working and active when her first grandchildren were born, “by the time the next tranche came along, the middle ones and the wee ones ... I’ve been doing it just enough to really enjoy them, but I am exhausted with it, I really am.” This shows the ambivalence of caring for (great)grandchildren but also the processual nature of the relationship as one that is lived through time as part of the aging process.

Participants also expanded understandings of intergenerational relationships to include ancestors and descendants. Duine (71, G1) interpreted the decreased life expectancy of children in *Last Children of Tokyo* as “a message to society that when you live for today and to hell with consequences for tomorrow, it is building up untold harm for your children, grandchildren and following generations.” Jamie (29, G3) echoed this, arguing that “the author has created this scenario as a kind of parable about each generation’s responsibility over the generations to come. [It’s] not as simple as living innocently and then being cared for in your old age but living with a radical responsibility.” Frankie (60, G1) also felt that “maybe we should be looking back, it’d be interesting to look back seven generations and say, ‘right that’s what I need to be making amends for’ or you know ‘what happened seven generations back from me, what horrors were carried out’ that might give a different perspective about it.” The novel *Sixteen Trees of the Somme* uses the genre of a family saga to grapple with this exact question, as Edvard searches for meaning in the past 100 years of family history to decide the future of his farm. The novel prompted differing responses in our readers. Jamie (29, G3) reflected, “personally, I think that we owe more to future generations than our ancestors, because they are the ones who are going to inherit the world.” Sanny (65, G3) added that:

we have a responsibility to our ancestors as well as to future generations. Our ancestry is a major player in who we are. Values are passed down from generation to generation. It can sometimes take a considerable time before we realise this but that does not detract from it being a truth.

This implies an awareness of change and continuity in intergenerational relationships, where it is possible to identify different generations while acknowledging how each is shaped by, and shapes, others. For participants, intergenerational relationships included familial, social, and public relationships happening *now* but also placed generations in dialogue with ancestors in the distant past and descendants in the near future. This perception of intergenerational relationships as one of mutual concern and responsibility occurring over several human lifetimes diverges from Scheffler’s argument of a contemporary “temporal parochialism” (Scheffler, 2018). The participants’ temporal framing of intergenerational relationships also runs contrary to the binary, antagonistic generationalism represented in contemporary dominant discourses, and is instead more akin to the longer-term, reciprocal relationships rooted in “environmental time” as articulated in Indigenous discussions of aging (Chazan & Whetung, 2021). Commitments to future generations, in particular, were evident in the emotional responses to the novels, which we discuss next.

### Ambivalent Emotions That Complicate the “Conflict/Solidarity” Binary

Several novels prompted discussions of the environment, and participants of all ages acknowledged emotions of guilt, responsibility, and blame, relating them to intergenerational relationships. This is unsurprising considering that the climate crisis is typically framed as an issue of “intergenerational justice” (Diprose & Valentine, 2019). However, participants articulated these emotions in more complicated and ambivalent ways than attributing blame and victimhood to discrete generations in a zero-sum fashion.

First, many of the older participants felt annoyed with what they perceived as being personally blamed for environmental crises. For example, *Trouble with Lichen* prompted discussions of the environmental impact of extended life expectancies. Christine (53, G3) felt she was being judged by her family on the topic: “I mean, even my daughter was saying ‘oh it’s you that’s ruined everything’ personally! ... so already there’s a bit of blame going on.” Discussing *Last Children of Tokyo* Rebecca (48, G2) similarly expressed that her children were also blaming her for “ruinin[g] the planet.” Although some of the older participants expressed some guilt and responsibility, they contextualized this within the socioeconomic, political, and technological practices and innovations experienced during their life course. Christine (53, G3) said “I’m far more aware of the climate emergency, I am more keen to do things ... I utilise things, trying to stop using plastic ... I don’t think that [my] generation is responsible for all the plastic waste per se ... but then that was the scientists at the time invented that and we all used it thinking that it was okay.” Greta (70, G3) agreed with this, saying “I always have this argument with people when they start talking about plastic and I’ll go “excuse me, not my generation ... we get the sort of label, you’re the previous generation so you must be responsible for all of that.” Participants used the discourse of generations, but stressed the necessity to understand individual action within wider social practices.

Second, participants of different ages invoked a temporal aspect to guilt; rather than seeing it as the preserve of particular age groups or generational cohorts, they projected themselves into the past or future, to consider what else they could have done—or could do—differently to take more responsibility. One example of this is a discussion about “generational guilt” in Group 3, which was prompted by the environmental dystopian setting of *The Last Children of Tokyo.* Clare (23, G3) began by saying “I think of when I’m older and there’s younger generations, in my head I’m like ‘they won’t be resentful towards me, I’ve done my bit’ but then ... like, I am very much susceptible to causing climate change, but I do think that element of guilt will always be there.” Responding to this, Jen (40, G1) said:

when we were seeing all the climate change marches that were going on, cause they were kind of the next generation down from me and I feel really guilty that I didn’t do enough when I was their age. When I was younger it was all about the ... hole in the ozone layer ... and stuff like that, and it kinda felt like that’s the only thing we concentrated on and then once we’d solved that ... we stopped doing anything else ... So I think there’s definitely a generational element to that guilt’

Kirsty (36, G1) agreed to some extent, although believed that:

the generations that came before us that developed the technology to have everything disposable ... did so with the right reasons ... so I can see why there would be guilt but ... it’s up to us what we do with the technologies and the products that we have and I don’t think we’ve made the right choices; I don’t think that’s my mum and my grandparents’ fault.

Discussions such as these were common across the groups. Elizabeth (60, G3) had a similar reaction to Jen and resisted the idea that “her generation” was ignorant of the environment: “in the circles I was in it was very much ... about the environment, climate, Rachel Carson and *Silent Spring* ... protests of all sorts.” However, she also felt that “it’s kinda like then we got to a certain level and everybody went ‘ooh, we could have a holiday now, time for champagne and opera’.” Jonathan (21, G2) thought that the characters in *The Last Children of Tokyo* were just resigned to a state of hopelessness [and…]it felt like there was a massive amount of guilt on the part of Yoshiro. Reflecting on the novel, he projected himself into the future, stating, “I’m going to be one of the older people so I’m going to be the one feeling guilty even though now I feel like I’m a younger person who gets to ... be like ‘well it wasn’t my fault’ ... and I guess it just goes to prove a point that I don’t think guilt is a very helpful reaction to it.”

A temporal dimension was also expressed in some participants’ invocation of an understanding of generations in a broader sense, akin to that of the “ancestors and descendants” understanding discussed in the previous theme. Jess (30, G4) found *The Last Children of Tokyo* “shocking” for its clarity in describing a world in which a child might only know of animals through his great grandfather’s memories. She said of Mumei:

him drawing all of the animals and never getting the opportunity to see them, it just reallybrought home quite what we’re doing to the planet and that that could be our future and I don’t think we’re quite ready in this generation to admit it. I don’t think we’ve all, everyone that’s alive now, has really accepted that climate change is a real thing.

Here she implies a definition of generation as “everyone that’s alive now” and in doing so, suggests a shared responsibility, regardless of age. This was also suggested by Jamie (29, G3) who argued “I think that if we are going to make any kind of advance on this then there needs to be a rallying together rather than throwing stones at each other.” This can be interpreted as a desire for solidarity in the face of conflict, but we argue that the lengthy discussions devoted to generational guilt and other emotions complicate this and reveal underlying tensions that cannot necessarily be resolved in a desire to address environmental disaster.

### Authenticity and Ambivalence in Fostering Intergenerational Contact

Participants welcomed relationships between people of different ages in the books and regarded intergenerational interaction as something to be valued and encouraged. However, they resisted what they saw as “contrived” attempts to bring people of different ages together. Instead, they called for “authentic” intergenerational engagement. This was prompted particularly when discussing *The Summer Book* and *Young Art and Old Hector*, both of which foreground the relationship between older and younger characters.

Set in the Highlands of Scotland in the first half of the twentieth century, *Young Art and Old Hector* by Neil Gunn (1941) tells the story of 8-year-old Art and his older neighbor, Hector. Participants loved the depiction of the relationship for what they perceived as its warmth and empathy between people across age groups. Kitty (70, G4) praised the novel for showing “life through an older person’s eyes and a young person, it was just that much clearer and I just felt like it just fitted somehow, you know, Hector’s experience, as if he ... really could connect with [Art], ... it felt like he could see easily the issues there for the kid.” Angela (39, G4) agreed: “I think it’s just a lovely relationship, that both of them really valued.” Participants across the groups reflected on the relationships in the novels in the context of their relationships with younger and older people in family and wider networks and spoke of the value of these experiences. Agnes (58, G4) recounted her own experiences of working with children and older adults in schools and the joy she took in this:

I’ve got one photograph in particular of a lady who was in her eighties ... talking to a child of ... about eight or nine, and the look between the eyes of these individuals is a connection over all these years talking about school teachers, and to me that’s the magic of it, it doesn’t matter how old you are, you’ve got a shared experience.

Although participants in all groups felt that these relationships were important for fostering empathy and learning across different stages of the life course, several participants felt that these interactions were rarer, for reasons including increased geographical mobility and a lack of time outside work and education. They called for new policies and practices that could encourage intergenerational relationships, focusing in particular on a desire for more “free time.” However, participants also articulated an ambivalent desire for authentic relationships; they desired authentic community connection, free from political interference, at the same time as they articulated a need for new policies that would free up time and space to build such communities.

Participants offered practical suggestions on how to use time more effectively. Kirsty (36, G1) felt having more time could encourage friendships across age groups and she wondered “if a younger retirement age might change that if there was a wider span of people enjoying the same activity together?.” This type of shared activity across ages was a motif of *The Summer Book*, where many readers across different groups found the grandmother and Sophia’s time together enviable, as they are free to stay curious and organize their own time on the island. The novel inspired them to imagine new possibilities for organizing time differently. Jonathan (21, G2) translated this desire for time into a potential policy solution: “we have the resources for, like, 3- or 3-day weeks for everyone and I think that would massively free up people to do what Sophia and her grandmother are doing which are, you know, exploring and playing and being creative [together].” Elizabeth (60, G3) suggested that a Universal Basic Income (UBI) would free people up to do more voluntary work or even change careers across their life course where “some might power on, some might want to change direction.” Alongside desiring more time, a desire for more accessible “intergenerational” space was also important to participants. Responding to *The Summer Book*, Eleanor (62, G4) wrote in a diary entry:

Do not keep elders and children apart, compartmentalising their experiences into age-appropriate ghettos, even if at times the relationship seems fraught or indifferent. Living and learning together improves our understanding of each other, builds stronger relationships, and teaches compassion.

Participants were aware of existing projects that sought to bring different generations together, including intergenerational co-living spaces, nursery, and care home exchanges and integrated dementia villages. The space of the island in *The Summer Book* was seen to naturally encourage intergenerational connection, prompting Jonathan’s (21, G2) idea to build a care home: “something that’s kind of a lot more open to the community where people can just interact rather than kind of you only visit a home if you’re visiting a relative,” Participants imagined new spaces where intergenerational mixing could happen, with calls for community centers to be reopened and devolved to communities.

Equally, participants said that they did not want these spaces to be “contrived” or “inauthentic.” The relationships between characters such as the grandmother and Sophia or Art and Hector were praised for being “genuine.” Reflecting on *Young Art and Old Hector*, Jane (69, G1) shared a story about a local man who was a stalwart in her community. Once he moved to a care home, he became more isolated and what would have been a natural, everyday occurrence of seeing his neighbors became reduced to visits that were “something you have to arrange.” Katherine (76, G2) also craved more “real example[s] of directly interacting with the [older] generation” such as intergenerational house sharing, where people would be “not forced to but encouraged to mix and mingle.” Angela (39, G4) cautioned against coercing participatory involvement, noting “you ’an’t force someone to take part in something, you know, that takes away what ’ou’re trying to do I think. Th’re’s only so much you can do, and it’s about sometimes focusing on what groups are a success […] Not everything’s going to be a success and it hasn’t been, but I think you’ve just got to try haven’t you?” Participants hoped community would naturally occur and lead to active, intergenerational communities based around reciprocity, shared values, and sincere connections.

## Discussion and Conclusion

In this paper, we have shown how fiction can be used to humanize and add nuance to the term “intergenerational relationships.” Notably, the novels read by the groups did not explicitly mention intergenerational relationships and made no reference to cohorts such as “boomer” or “millennial.” Instead, they presented readers with complex, emotional, and ambivalent examples of relationships between people of different ages that readers could relate to. The paper makes a number of key contributions to how intergenerational relationships can be engaged with more meaningfully, beyond simplistic and reductive dichotomies. First, it expands dominant definitions of intergenerational relationships beyond “fixed, cross-sectional cohorts” of people of different ages alive today, to include relationships that are lived and which transition over time. Second, it identifies greater complexity and ambivalence in the articulation of emotions relating to generations and generational identities relating to environmental crises than is depicted in “conflict/solidarity” discourses. Third, it presents ambivalent desires for, on the one hand, policies that foster greater “meaningful intergenerational interaction,” while cautioning against interventions that are “contrived” and “inauthentic.”

The paper contributes to gerontological understandings of intergenerational relationships and ambivalence through its use of fiction and reading groups. Existing scholarship has identified ambivalence as a useful concept in “resolving rivalry and ... moving beyond generational oppositions” (Biggs, 2007, p. 706) and to circumvent conflict/solidarity binaries (Lüscher & Pillemer, 1998). By using a methodology of readers’ responses to novels, we show how fictional representations of intergenerational themes can elicit more meaningful reflections on the ambivalences and complexities of relationships across age groups. Notably, the ambivalence expressed by participants was rooted and revealed in reflections on their own lived experiences and their reactions to relatable fictional characters and scenarios. This is closer to the “lived ambivalence” that Baars (2014 invokes). Although Baars used the term more in relation to aging, rather than specifically intergenerational relationships, the experiential, temporal, and processual dimensions invoked by the word “lived” help us to make sense of the findings presented here. We endorse Baars’ insistence that time be given more attention to understanding the aging process but extend this to specifically include intergenerational relationships. In reflecting on the novels, participants spoke about intergenerational relationships as ambivalent processes lived in time. This temporal dimension was evident in participants’ discussions about past, existing and anticipated caring responsibilities, extended definitions of intergenerational relationships as encompassing “middle generations” and ancestors and descendants, personal regrets, and anticipated guilt contextualized within historical processes, and a desire for more time to allow for “authentic” intergenerational connection.

In addition to showing how fiction can deepen understanding of gerontological issues, we suggest that our paper also offers insights into how gerontological concerns can inform interpretations of fiction. Although we argue that the very absence of terms such as “intergenerational relationships” or “intergenerational segregation” in the novels freed participants to express more nuanced and ambivalent thoughts than might otherwise have been the case, their awareness of these issues—in part, but not wholly prompted by our questions—guided their reading. Whether in speculative fiction such as *Trouble with Lichen* or *The Last Children of Tokyo*, or more realistic representations in *Young Art and Old Hector* and *The Summer Book*, participants responded enthusiastically and ably to our invitation to read them with a “gerontological lens” and were well versed in the vocabulary of dominant discourses of intergenerational conflict, inequality and justice in their discussions, even as they expressed more ambivalence. These interdisciplinary pathways between gerontological concerns and imaginative reader responses suggest that both approaches are needed to develop new ways of thinking through the complexities of intergenerational relationships and the transitional experience of living/aging through time.

A number of policy implications arise from this study. Given the marked ambivalence expressed by participants across different subjects and their desire for “authenticity,” we agree with Biggs’s (2007) suggestion that policymakers would be wise to embrace ambivalence to “acknowledg[e] and negotiat[e] solutions promoted between generational groups” (pp. 706–707). Participants echoed concerns about age segregation (Hagestad & Uhlenberg, 2006) and, valuing what they perceived as the “genuine” intergenerational connections depicted particularly in the novels *The Summer Book* and *Young Art and Old Hector*, called for more time and space in being allowed to develop these relationships. The ambivalence expressed here was one where participants wanted policymakers to facilitate greater intergenerational connection, without necessarily labeling interventions as such. Accordingly, the policies they endorsed were not explicitly targeted at addressing age divisions. Therefore, organizations that work with intergenerational groups might consider how present policies currently being trialed such as the 4-day week (Cooper et al., 2021; Haraldsson & Kellam, 2021), UBI (Ugo et al., 2020), urban design (UN-Habitat, 2020), and climate action (UNEP, 2021) intersect with lived experiences of aging and what intergenerational communities can contribute to these ideas.

This paper addresses calls to “myth-bust” generational conflict (Duffy, 2021) but also calls for nuance and complexity in invoking intergenerational solidarity. The findings presented in this paper are underpinned by a methodology that uses fiction to provide a platform for ambivalent discussions and feelings, where novels can open up new worlds and allowing for recognition to “forge connections across difference” (Felski, 2019) and offer new vocabularies for discussing intergenerational relationships. In doing so, we offer a way to avoid the pitfalls of a flattening “generationalism” (White, 2013) and bring more insight into what intergenerational relationships are and why they matter.

## Supplementary Material

gnad051_suppl_Supplementary_Material

## Data Availability

For the purpose of open access, the authors have applied a Creative Commons Attribution (CC BY) license to any Author Accepted Manuscript version arising. The data supporting the findings reported in this paper will be available via the UK Data Archive repository from April 2023. This study was not preregistered.
