# Supplementary material for: Understanding Nuance and Ambivalence in Intergenerational Relationships Through Fiction
Source: Gerontologist. 2023 Apr 27;63(10):1619–27. doi: 10.1093/geront/gnad051 (PMC10724047; doi:10.1093/geront/gnad051)
Supplement: gnad051_suppl_Supplementary_Material [file gnad051_suppl_supplementary_material.docx]

**Online Supplementary Material**

Supplementary Table 1: Information on novels and the generational relationships and themes foregrounded in them

| **Title** | **Author** | **Date of first publication** | **Genre** | **Generational relationships/themes foregrounded** |
| --- | --- | --- | --- | --- |
| The Summer Book | Tove Jansson | 1972 | Realist | Grandmother and grandchild  Father and daughter  Mother and son |
| Trouble with Lichen | John Wyndham | 1960 | Science / speculative fiction | Future generations, increased life expectancy |
| The Dark Flood Rises | Margaret Drabble | 2016 | Realist | Mother, adult daughter |
| The Last Children of Tokyo | Yoko Tawada | 2014 | Science / speculative fiction | Great-grandparent,  Great-grandchild |
| Young Art and Old Hector | Neil Gunn | 1941 | Realist / historical | Older mentor,  Child |
| Turnabout | Margaret Peterson Haddix | 2000 | Science / speculative fiction. Young adult. | Future generations,  increased life expectancy |
| Moon Tiger | Penelope Lively | 1987 | Realist/ historical | Ancestors,  Family networks,  Mother and adult daughter |
| Never Let Me Go | Kazuo Ishiguro | 2005 | Science / speculative fiction | Future generations |
| The Sixteen Trees of the Somme | Lars Mytting | 2014 | Realist/ historical | Grandfather and grandchild. Great-uncle and great-nephew. |
